# The influence of tumor oxygenation on ^18^F-FDG (Fluorine-18 Deoxyglucose) uptake: A mouse study using positron emission tomography (PET)

**DOI:** 10.1186/1748-717X-1-3

**Published:** 2006-02-28

**Authors:** Linda W Chan, Sebastien Hapdey, Sean English, Jurgen Seidel, Joann Carson, Anastasia L Sowers, Murali C Krishna, Michael V Green, James B Mitchell, Stephen L Bacharach

**Affiliations:** 1 Radiation Oncology Branch, Center for Cancer Research, National Cancer Institute, National Institutes of Health, Department of Health and Human Services, Bethesda, MD, USA; 2 Department of Nuclear Medicine, Clinical Center, National Institutes of Health, Bethesda, MD, USA; 3 Radiation Biology Branch, Center for Cancer Research, National Cancer Institute, National Institutes of Health, Department of Health and Human Services, Bethesda, MD, USA

## Abstract

**Background:**

This study investigated whether changing a tumor's oxygenation would alter tumor metabolism, and thus uptake of ^18^F-FDG (fluorine-18 deoxyglucose), a marker for glucose metabolism using positron emission tomography (PET).

**Results:**

Tumor-bearing mice (squamous cell carcinoma) maintained at 37°C were studied while breathing either normal air or carbogen (95% O_2_, 5% CO_2_), known to significantly oxygenate tumors. Tumor activity was measured within an automatically determined volume of interest (VOI). Activity was corrected for the arterial input function as estimated from image and blood-derived data. Tumor FDG uptake was initially evaluated for tumor-bearing animals breathing only air (2 animals) or only carbogen (2 animals). Subsequently, 5 animals were studied using two sequential ^18^F-FDG injections administered to the same tumor-bearing mouse, 60 min apart; the first injection on one gas (air or carbogen) and the second on the other gas. When examining the entire tumor VOI, there was no significant difference of ^18^F-FDG uptake between mice breathing either air or carbogen (i.e. air/carbogen ratio near unity). However, when only the highest ^18^F-FDG uptake regions of the tumor were considered (small VOIs), there was a modest (21%), but significant increase in the air/carbogen ratio suggesting that in these potentially most hypoxic regions of the tumor, ^18^F-FDG uptake and hence glucose metabolism, may be reduced by increasing tumor oxygenation.

**Conclusion:**

Tumor ^18^F-FDG uptake may be reduced by increases in tumor oxygenation and thus may provide a means to further enhance ^18^F-FDG functional imaging.

## Background

^18^F-FDG (fluorine-18 deoxyglucose) has become widely used as a radiolabeled marker for positron emission tomography (PET) imaging of solid tumors. In some regions of the body, the predictive power for identifying cancer using ^18^F-FDG approaches 95% [1,2].

While altered glucose metabolism is a unique feature of neoplastic growth, there are other factors associated with the tumor micro-environment that are in marked contrast to normal tissues. The vascular architecture in tumor tissue is abnormal and differs greatly from normal tissues, resulting in altered blood flow and the development of tumor hypoxia. The presence of tumor hypoxia is thought to represent a barrier for effective cancer treatment for both radiation and chemotherapy [3-6]. Identifying patients whose tumors contain hypoxic areas may therefore have an important role in tumor prognosis as well as treatment approach and outcome. Currently, the ability to identify and quantify tumor hypoxia is limited. The current gold standard for measuring tissue oxygen concentration utilizes oxygen-sensitive electrodes, which measure oxygen partial pressure (pO_2_) directly in tumor tissue. Given the invasive nature of this technique, it is difficult to access deep-seated tumors, and once assessed, it is difficult to distinguish between measurements made in necrotic and viable regions [7]. Non-invasive techniques, such as Overhauser-enhanced magnetic resonance imaging (OMRI) [8] and electron paramagnetic resonance imaging (EPRI) [9,10], are being evaluated to avoid the obstacles encountered with the polarographic electrode.

Since non-invasive ^18^F-FDG/PET imaging is already widely used in clinical facilities to identify malignant tissue, we questioned whether this imaging modality might be used to assess tumor hypoxia. Anaerobic metabolism requires much more glucose to generate the same amount of ATP as under aerobic metabolism. Given the hypoxic nature of certain tumors, regions of tumor tissue are known to resort to anaerobic glucose metabolism [11]. We hypothesize that experimentally increasing a tumor's oxygen supply might enable the tumor to metabolize more glucose aerobically, thus reducing ^18^F-FDG uptake. To test this hypothesis, we have compared tumor ^18^F-FDG uptake in tumor-bearing animals sequentially breathing air (20.9% O_2_) and carbogen (95% oxygen, 5% CO_2_). Carbogen breathing has been shown to markedly increase the oxygenation status of tumors [8,12]. In addition, we present data stressing the importance of maintaining normal body temperature in small animals undergoing ^18^F-FDG/PET imaging.

## Results

**Figure 1:**
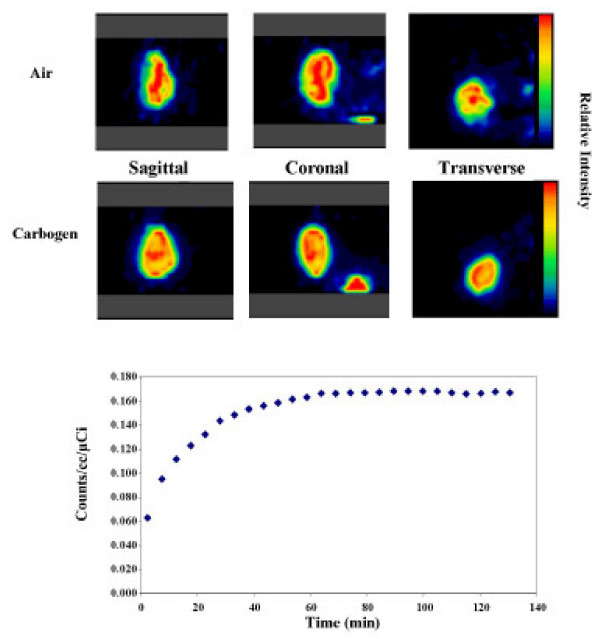
^18^F-FDG images (top) showing sagittal, coronal, and transaxial slices through the tumor of a mouse (maintained at 37°C) breathing air and a different mouse breathing carbogen. Both color scales set to the same maximum nCi/cc/injected-dose. Bottom: time activity curve obtained from the VOIs drawn around the entire tumor for a tumor-bearing animal breathing air (animals breathing carbogen also resulted in similar curves, flat at late times).

Figure 1 shows images from two of the pilot studies. Figure 1 (top) shows sagittal, coronal, and transaxial slices through the tumor of a mouse (maintained at 37°C) breathing air and a mouse breathing carbogen. Figure 1, bottom, shows the time activity curve obtained from the VOIs drawn around the entire tumor for a tumor-bearing animal breathing air. The ordinate of the time activity curve is the relative ^18^F-FDG activity concentration in the tumor per mCi injected dose. Note that the decay corrected FDG uptake curve is quite flat from 60 min until the end of the study (130 min). This was true for all four mice in the pilot study. Similar time activity curves were obtained for animals breathing carbogen (data not shown). Note also that the uptake in these two mice (one on air and one on carbogen) was visually similar with considerable heterogeneity of ^18^F-FDG uptake across the tumor. Air versus carbogen comparisons could not be made for these animals however, because data for the mice in the pilot studies could not be corrected for possible differences in the arterial input function. In addition, ^18^F-FDG uptake would presumably have mouse-to-mouse physiologic variation since different mice were used for air and for carbogen, potentially masking true differences in uptake caused by the nature of the ventilating gas. For these reasons the second more elaborate set of studies described above were undertaken. This second set of studies involved two sequential ^18^F-FDG injections in the same mouse, 60 minutes apart, the first injection on one gas (air or carbogen) and the second on the other gas. The fact that the ^18^F-FDG time activity curves from the pilot studies were flat, regardless of whether air or carbogen was used, meant that in the two injection studies, we could subtract the ^18^F-FDG activity present at the end of first 60 min from the activity following the second injection. These two injection studies permitted sequential measurement of air and carbogen uptake in the same tumor, and permitted correction for possible differences in the arterial input function.

### Air to carbogen uptake ratios at normal temperature (37°C)

**Figure 2:**
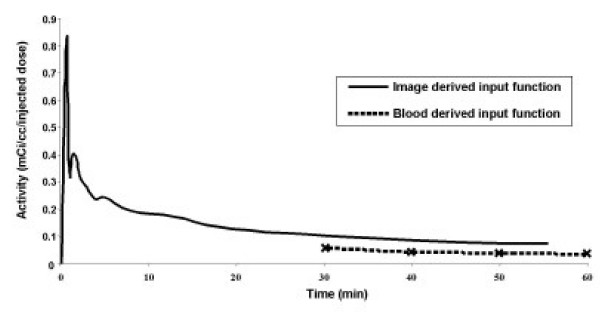
Blood- and image-derived input functions for mice scanned at normal physiologic core body temperature (37°C). Blood-derived input functions were determined using blood samples drawn every 10 min, starting 30 min after ^18^FDG injection. Thus, the blood-derived input function curve begins at time = 30 min. Note that the blood samples always yielded lower activity concentration than the image data due to background activity, as per equation 1.

**Figure 3:**
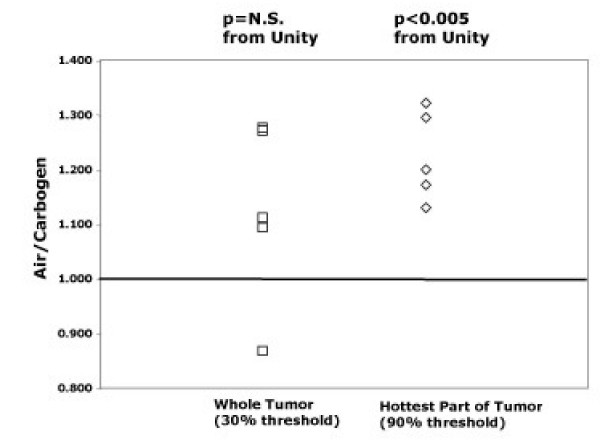
Air/carbogen ratios of tumor ^18^FDG uptake for whole tumor VOI (squares), as determined with the 3D region growing program using a 30% threshold, and for the hottest part of the tumor (90% threshold). Tumor uptake ratios were corrected using derived input function data as per equation 2 and the 2^nd ^injection activity was corrected for residual activity remaining as a result of the first injection.

For each mouse we computed the ratio of the uptake while breathing air to the uptake while breathing carbogen, each corrected for the area under the input function as per equation 2. A typical input function is shown in Figure 2, along with the late blood sample derived input function. The measured input function was corrected using the blood samples, as per equations 1–3. The ratio of corrected tumor uptake while breathing air to corrected tumor uptake while breathing carbogen, averaged over all 5 mice, did not differ significantly from 1.0 (ratio air/carbogen = 1.15, p = NS, Figure 3, squares). The ratio remained not significantly different from unity whether the VOI was drawn using a 30, 40 or 50% threshold, and even when manually drawn VOIs encompassing the entire tumor were used. Note from Figure 3 that the tumors of four out of five mice exhibited air/carbogen ratios, all >1, while one mouse deviated from the other 4, with a ratio lower than unity. There was no *a priori *reason to exclude the outlying animal, but doing so resulted in an air/carbogen ratio, which did differ significantly from unity (air/carbogen = 1.22, p < 0.01).

**Figure 4:**
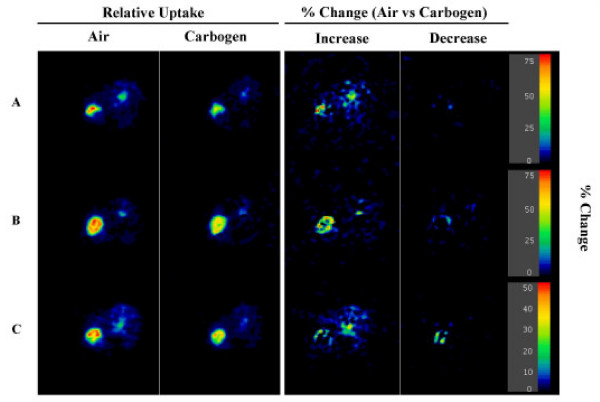
Sagittal ^18^FDG tumor uptake images for three mice (A, B, C) receiving two injections of ^18^FDG, one while the animal was breathing air and the second after the animal was switched to carbogen breathing, or vice-versa (animals maintained at 37°C throughout the study). The first column shows the relative tumor ^18^F-FDG uptake for air breathing. The second column represents tumor FDG uptake for the animal after switching to carbogen breathing (residual ^18^F-FDG from initial injection subtracted). The first and second columns are shown with the same relative color scale. The third column shows the pixels in which air uptake exceeded carbogen uptake, expressed as 100*(AirImage - CarbogenImage)/(mean counts in Air tumor VOI). Ideally the denominator would have been the air image, but due to image noise considerations the normalization described was done instead. Negative values are not displayed in column 3. Instead the fourth column shows the (many fewer) pixels in which carbogen exceeded air (i.e. the negative of the equation used for column 3. Corrections were made for injected activity and for residual activity. The numeric values shown on the color bar apply only to columns 3 and 4.

As shown in Figure 1 the SCC tumor exhibited considerable heterogeneity in its uptake of ^18^F-FDG. We hypothesized that regions of the tumor demonstrating accentuated uptake of FDG might be exceptionally hypoxic and so might respond differently to an increase in the concentration of inspired oxygen as compared to other tumor tissue. To test this hypothesis we analyzed small VOI's (90% threshold) around regions of the tumors demonstrating the greatest activity. Unlike the data from the whole tumor, the uptake (corrected for input function) for these small hot VOIs resulted in an air/carbogen ratio that was consistent across all 5 mice, and was significantly greater than unity (air/carbogen = 1.21, p < 0.005, Figure 3, right). Again this result was insensitive to the way the VOI was defined. The results held when the VOI was based on either an 80% or 90% threshold, or even if the maximum tumor value (averaged with its closest neighboring pixels in all 3 dimensions) was used for each tumor. We wished to visualize which pixels were causing the difference in ^18^F-FDG tumor uptake for air versus carbogen breathing. Figure 4 shows ^18^F-FDG tumor uptake images for three different animals who received two ^18^F-FDG injections, one while breathing air and the other while breathing carbogen. The first column shows the tumor ^18^F-FDG uptake for air breathing. The second column represents tumor ^18^F-FDG uptake for the animal after switching to carbogen breathing (residual ^18^F-FDG from initial injection subtracted). From these images it is difficult to tell which pixels were causing the slight decrease in tumor ^18^F-FDG uptake for animals breathing carbogen. To clarify this, the third column shows the percentage increase in ^18^F-FDG uptake for air breathing animals versus carbogen breathing animals expressed as 100* (AirImage – CarbogenImage)/(mean counts in Air tumor VOI). Ideally the denominator would have been the air image, but due to image noise considerations the mean value in the air tumor VOI (30% threshold) were used instead. The data were normalized so that when the tumor VOI was placed on the percentage increase image, it gave a value equal to the value shown in the corresponding point in Figure 3. Negative values are not displayed in column 3. Instead the fourth column shows the (many fewer) pixels in which carbogen exceeded air (i.e. the negative of the equation used for column 3).

### Air to carbogen uptake ratios at low temperature (30°C)

**Figure 5:**
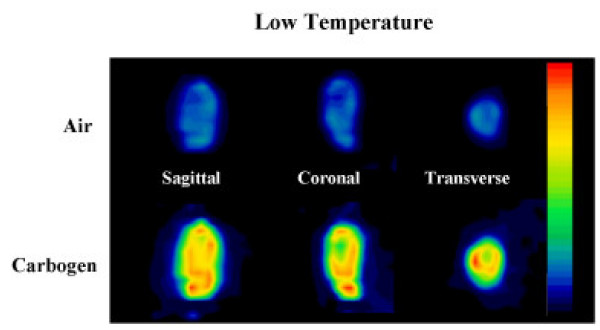
^18^F-FDG images showing sagittal, coronal, and transaxial slices through the tumor of a mouse maintained at 30°C given two injections of ^18^F-FDG, the first while breathing air and the second while breathing carbogen. Both images are displayed to the same scale, and are corrected for injected activity and residual activity produced by the first injection.

When the core temperature of the mice was maintained at 30°C, an air/carbogen tumor ratio (averaged over all mice) very much less than one was observed (mean air/carbogen ratio = 0.47 ± 0.30; n = 2). Again, this result was insensitive to the manner of VOI definition. Figure 5 shows ^18^F-FDG uptake during carbogen and air for a low temperature mouse. It is quite clear that the tumor uptake while breathing carbogen is markedly higher than the uptake while breathing air. However, whereas the blood-derived input functions from mice at normal physiologic temperature (37°C) were highly reproducible across multiple mice, there was a large variability between blood-derived input functions in the low-temperature mice. Thus, one must interpret the data from mice not maintained at physiologic temperature with caution.

## Discussion

Hypoxia in human tumors can significantly influence treatment outcome and the aggressiveness of the tumor [13]. This finding has led to considerable interest in non-invasive means to assess the extent of hypoxia in tumors prior to therapy, for example with MR (BOLD) imaging [14,15]. Several studies have shown that tumor cell or tumor ^18^F-FDG uptake is associated with hypoxia [16-18]. Glucose transporter (GLUT) receptor proteins and hexokinase activity are elevated in tumors and thought to be responsible for increased ^18^F-FDG tumor uptake compared to normal tissues [19]. Hypoxia has been shown to be a significant factor in the over-expression of these proteins [16,17,20]. In the present study we examined whether information concerning hypoxia could be gained using ^18^F-FDG and PET imaging. This was considered possible since the hypoxic nature of most tumors forces tumor glucose metabolism to utilize primarily glycolytic pathways, rather than oxidative metabolism [11]. Very large quantities of glucose are required to produce a given amount of ATP via the glycolytic pathway compared to the oxidative pathway, which supposedly accounts for why many tumors have such high ^18^F-FDG uptake. We hypothesized that by oxygenating the tumor (via breathing carbogen), tumor glucose metabolism would begin to have a significant oxidative component, requiring considerably less glucose per ATP molecule produced. Thus, better oxygenated tumors (i.e. tumors influenced by breathing carbogen) should have less ^18^F-FDG uptake than when in their usual, hypoxic state (i.e. while the animal is breathing air). To examine this possibility, ^18^F-FDG uptake in tumors was examined in tumors when the mouse was breathing air versus carbogen.

The SCC tumor used in the present study is known to be quite hypoxic as determined by previous oxygen electrode measurements in our laboratory [8]. Even though the tumor is extremely hypoxic, there was no evidence of necrosis in the tumors used in the present study (~1 cm diameter). Carbogen breathing has been shown in numerous studies to increase the oxygenation status of tumor-bearing mice [8,21] and in patients [22-24]. Using the same SCCVII tumor model, tumor size, and experimental conditions with respect to the maintenance to core body temperature as used in the present study, we have previously shown that carbogen breathing increases oxygenation of the SCCVII tumor as measured by Eppendorf oxygen electrodes [8,8]. In this study the mean tumor pO_2 _values for animals breathing air was 8.2 mm Hg, which increased to a value of 19.8 for animals breathing carbogen [8].

As mentioned above, the hypoxic nature of the SCC tumor makes it likely that a large proportion of the tumor cells would utilize glucose by anaerobic glycolysis, and so exhibit enhanced ^18^F-FDG uptake. Indeed, we found glucose utilization as measured by uptake of ^18^F-FDG to be significant in the SCC tumor as evidenced by the tumor images shown in Figure 1. The FDG uptake in the tumor was particularly striking compared to the non-tumor bearing leg where little to no uptake was observed (data not shown). It was anticipated that increasing the oxygenation of the tumor by carbogen breathing would lead to a shift to aerobic metabolism producing much more ATP per molecule of glucose, thereby decreasing the need for glucose, and decreasing ^18^F-FDG tumor uptake. The results of this study only partially supported this hypothesis. Air/carbogen ratios for overall tumor ^18^F-FDG uptake were not significantly different from unity. However, when only the highest ^18^F-FDG uptake region of each tumor was considered (perhaps the most hypoxic regions) the hypothesis was supported. In these presumably metabolically active regions, there was a small (21%) but significant increase in air/carbogen uptake, suggesting reduced ^18^F-FDG uptake under conditions of better oxygenation. The lack of an air/carbogen difference for the whole tumor, and the fairly small air/carbogen difference even for the hottest part of the tumor might suggest that tumors are "programmed" to burn glucose primarily in an anaerobic fashion, regardless of their level of oxygenation. This could be a potential survival mechanism for tissue destined to grow in an anaerobic environment. This potential explanation is of course speculative. Another factor which could play a role is the potential vasodilatory properties of carbon dioxide, although there is conflicting data reported in the literature [24].

Changes in tumor FDG uptake (hot spots) when breathing carbogen might add another dimension to ^18^F-FDG functional imaging. While small, the 21% change in ^18^F-FDG uptake in small regions may be giving information similar and perhaps complimentary to that obtained by the MRI based oxygen imaging is Blood Oxygen Level Dependent (BOLD) MRI. For this imaging technique, temporal changes in the ratios of deoxy- to oxyhemoglobin can be monitored utilizing the contrast mechanisms provided by deoxyhemoglobin to protons. With this method, it is possible to examine changes in blood flow and tumor oxygenation levels in response to carbogen breathing versus air breathing [14,15]. The enhancement observed in BOLD MRI images is typically only a few percent. In contrast, the decrease in ^18^F-FDG tumor uptake upon carbogen breathing in small regions of the tumor in the present study was up to 21%, suggesting that in these small regions tumor glucose metabolism is altered as a result of increased perfusion and oxygenation. Should this observation be correct, which will require further validation, the added functional dimension to ^18^F-FDG imaging afforded by carbogen breathing could be useful to clinicians assessing treatment response, particularly for radiation treatment.

Our results did not wholly agree with some previous studies that showed whole tumor ^18^F-FDG uptake increases in more hypoxic conditions [25-28]. Our whole tumor results gave an air/carbogen uptake ratio that did not differ significantly from unity. Only when VOIs were drawn around the hottest part of our tumors were our findings more consistent with these previous studies. The aforementioned studies either examined a different tumor type from our study (C3H mammary carcinomas) [25], or examined the same tumor type (SCC), but in an *in vitro *setting [26]. These differences alone could potentially account for the difference in our findings. Additionally, this result could be due to differences in experimental method, or due to the variability in our own results. Previous studies did not attempt to correct for potential differences in input function. The lack of standardization across animal PET studies, both in the means of determining reference input functions, as well as lack of consensus regarding simpler procedures such as controlling animal core temperature, might also explain this difference in result. Future experiments with a greater number of mice, using simultaneous blood sampling, would clarify the results shown in Figure 3.

The ability to examine and quantify regions of greatest change in ^18^F-FDG uptake, which is where a radiation oncologist would presumably concentrate three-dimensional conformal radiation dose painting, has significant implications for cancer diagnosis and treatment. Future studies could further test this phenomenon by shifting to more hypoxic conditions, to see if ^18^F-FDG uptake improves even more.

As an adjunct to our study, we examined the results obtained when mice were allowed to drop core body temperature to 30°C. Past work in our laboratory has demonstrated that animal's core body temperature under isoflurane anesthesia drops ten degrees in an average of 15 min [27]. We found that mice at this lower temperature showed significantly greater ^18^F-FDG accumulation in carbogen than air environments. This could be due to relative vasoconstriction in low temperature conditions, possibly making ^18^F-FDG uptake flow limited in these circumstances. Adding carbogen to this low-temperature state may improve blood flow, because vasculature responds to the high partial pressure of oxygen by vasodilation. Although this reasoning is speculative, our low temperature findings do point definitively to the necessity for strict control of the mice's physiology and the need for standardization across animal PET studies.

One strength of the present study was the ability to measure ^18^F-FDG uptake with air and carbogen breathing in the *same *tumor. Previous studies have compared uptake across different animals. Differences in tumor heterogeneity across mice potentially confound these results. Our study controlled for this confounding variable. Further studies would benefit from testing multiple tumor types, as well as human tumors.

## Conclusion

The ratio of ^18^F-FDG uptake in SCC tumors while breathing air to that while breathing carbogen, did not differ significantly from unity when the entire tumor was considered, and when the animal's temperature was kept at 37°C. When only the highest uptake regions of the tumor were considered, however, there was a modest (21%) but significant increase in the air/carbogen ratio. This suggests that in these potentially most hypoxic regions of the tumor, ^18^F-FDG uptake may be reduced by increases in tumor oxygenation and thus may provide a means to further enhance ^18^F-FDG functional imaging. As a secondary finding, we note that when the mouse's temperature was permitted to fall to 30°C (as occurs naturally during isoflurane anesthesia), significant alterations in ^18^F-FDG metabolism were observed, making it clear that strict physiological controls are necessary when such experiments are performed.

## Methods

### Animals and tumors

Female C3H mice, produced by the National Cancer Institute Animal Production Area (Frederick, MD), were used for this study. The mice were 7–9 weeks of age at the time of experimentation and weighed between 20–30 grams. All experiments were carried out under a protocol approved by the National Cancer Institute Animal Care and Use Committee, and were in compliance with the *Guide for the Care and Use Of Laboratory Animal Resource, (1996) *National Research Council. Tumors were grown in the mice by a subcutaneous (s.c.) injection of a single-cell suspension of 2 × 10^5 ^squamous cell carcinoma (SCCVII) cells in the right hind leg. Tumors grew to a size of ~1.0 cm diameter 7–10 days after injection. Tumor volumes were determined prior to PET scanning, by measuring three orthogonal diameters using calipers. For all imaging studies ~1 cm diameter tumors were used.

### PET scanner

PET images were obtained with the ATLAS (Advanced Technology Laboratory Animal Scanner), a dedicated small animal PET scanner developed at NIH with an axial field-of-view (FOV) of 2 cm, a transverse FOV of 6.8 cm and an aperture of 8 cm [28-30].

The image data acquired with the ATLAS PET scanner were reconstructed using the three-dimensional ordered subset expectation maximization (OSEM) reconstruction algorithm, including a model of the system resolution [31]. All reconstructions were performed on the NIH Biowulf computer cluster, utilizing sixteen subsets and taking 10 iterations. Under these conditions, a spatial resolution of 1.6 mm full-width-at-half-maximum (FWHM) was achieved.

### PET scan experimental set-up

The mice were fasted at least 3 hours prior to injection of ^18^F-FDG. Each mouse was anesthetized with isoflurane (induction; 2.0%, maintenance; 1.5%) carried by air at 750 ml/min and delivered via a nosepiece. An anesthetic delivery/scavenging device was continuously used throughout the experiment. Once anesthesia was established, further isoflurane was carried by either air (20.9% oxygen) or carbogen (95% oxygen/5% CO_2_). In order to inject^18^F-FDG intravenously (IV), a cannula was inserted into a tail vein. The IV lines consisted of a 30 gauge 1/2" needle inserted into one end of a 60 cm length of PE10 tubing (IntraMedic Polyethylene) (internal diameter = 0.28 mm; external diameter = 0.61 mm) and the needle of another removed from the hub inserted into the opposite end of the tubing. IV lines were filled with heparin (100 USP units/mL) prior to cannulation. To verify the integrity of the line, a bolus of heparin was given prior to ^18^F-FDG injection. A bladder catheter (PE10 tubing) was used to collect urine output and a rectal temperature probe (FISO Technologies Fiber Optic Temperature Gauge Model FOT C-PEEK) was used to monitor and maintain core body temperature. Once these procedures were completed the mouse was immobilized in a jig that secured the feet. This jig was inserted into a cylindrical Lucite chamber, fixed with a port for the attachment of thermostat-controlled, warm air circulator (Nikon Model ITC-32, Nikon Inc. Japan) in order to maintain the mouse's core temperature. The entire assembly was fixed to a computer-controlled motorized gantry capable of precisely moving the animal into the scanner. After core temperature of 37°C was established, PET image collection was started, and a 100 μl bolus of ^18^F-FDG (~400 μCi in 0.9% saline) was injected. The injected doses were recorded for each animal imaged. Initial pilot studies were performed on 4 mice by centering the tumor in the field-of-view prior to injection. Two of these first mice were studied only with carbogen and two only with air. For each mouse, a dynamic acquisition of 5 min/frame was acquired for 90 min total. In subsequent experiments each mouse was studied sequentially on both air and carbogen. These sequential studies, performed on an additional 5 mice were more complex, each consisting of 5 phases. Initially, each animal was anesthetized using either air or carbogen as the carrier gas for the isoflurane (3 with air and 2 with carbogen). While this gas was continuously administered, the scanner was positioned at the level of the heart, and dynamic imaging begun immediately prior to injection of ^18^F-FDG (12 frames/10 sec for 2 min, 6 frames/30 sec, followed by 45 frames/1 min), continuing for 50 min, in order to capture an image-derived input function. The scanner was then moved to the level of the tumor (the lower extremities), and dynamic imaging (5 min/frame) resumed for an additional 40 min, while continuing to administer the same mixture of isoflurane and either air or carbogen. At the end of this acquisition, the carrier gas for isoflurane was switched from air to carbogen, or vice versa, and the animal was left to equilibrate on the new gas mixture for 10 min. When the 10-minute equilibration period had ended, the scanner was again moved to the level of the heart and dynamic image collection commenced as described above, beginning approximately 30 sec prior to injection of a second bolus of ^18^F-FDG, first over the heart and then over the tumor. All the studies described above were performed while the mouse's body temperature was kept at 37°C.

An additional 2 mice were studied using this same two injection methodology but while their body temperature was kept at 30°C. We have previously shown that C3H SCCVII tumor-bearing animals placed on isoflurane experience an approximate 7°C decrease in core body temperature 15 min after initiation of isoflurane [27]. To conduct low temperature studies animals were prepared as described above and were administered isoflurane without temperature control until the core body temperature dropped to 30°C at which point the warm air circulator was adjusted to maintain a core body temperature of 30°C. This temperature was maintained throughout the two injection study.

### Blood sampling

After anesthetizing the mouse with isoflurane carried by medical air (1.5%, 700 mL/min), the skin and integument over the right or left ventral surface of the neck was cut with surgical scissors and the cleidomastoideus and sternomastoideus muscles were dissected to expose the left external jugular vein, removing fat and/or cauterizing peripheral vessels to minimize blood loss. A cannula, like that described previously for tail vein cannulation, was then inserted into the external jugular vein and fixed in place with Vetbond tissue adhesive. A junction was made by soldering a 30 G 1/2" needle into a 23 G 1" needle. The 30 G 1/2" needle end could be inserted into the end of the central line, while the 23 G 1" needle end could then be inserted into a small piece of PE50 tubing, into which a 10 μL Hamilton syringe could be inserted for a more precise collection of blood. After administering ^18^F-FDG (~400 μCi, 100 μl) bolus, 10 μL of blood were drawn from the central line every ten minutes for one hour. Each blood sample was then diluted in 1 mL of saline, and its activity was read in a Cobra II AutoGamma scintillation counter.

### Image-derived and blood-draw derived input functions

It is possible that the input function (the concentration of arterial ^18^F-FDG as a function of time) would change depending on whether the mouse was breathing air or carbogen. The area under the input function is a measure of the amount of ^18^F-FDG available for the tumor to metabolize. In order to compare air and carbogen ^18^F-FDG uptakes, the ratio of mean air to carbogen uptake in the tumor volume of interest (VOI) was computed, normalizing by the integral of the input function from time zero up to the time of the measurement of uptake. The input function was estimated and used for normalization as described below.

PET images at early time points revealed a large vessel dorsal and/or caudal to the heart. Comparison with CT images indicated that the vessel was the inferior vena cava, but this could not be determined with absolute certainty due to possible small mis-registrations between PET and CT. A strict determination of whether the vessel was arterial or venous was thought unnecessary, apart from any first transit uptake by the lung (known to be low), especially since the bolus was slow compared to the expected rapid cardiac transit times [32].

To determine a measured blood time activity curve, A_m_(t), using the major blood vessel, regions of interest were drawn around this vessel at several transaxial levels to determine the relative mean activity concentration in the vessel over time. Only relative concentrations could be determined because the diameter of this vessel was thought to be small compared to the spatial resolution of the scanner, making partial volume effects important. Furthermore, tissues adjacent to the vessel could have potentially contaminated the activity measured in the region of interest [33]. Corrections for these effects were made in the following way. A square region of interest (ROI), 2.8 mm on a side and encompassing the aorta was drawn and used to generate an aortic time activity curve. Two concentric rectangles were then drawn around this first ROI (7.3 mm and 9.6 mm on a side) and a background region was obtained by subtracting the smallest ROI from the largest ROI. Another time activity curve was created from this background region in order to estimate the background activity, B(t). It was assumed that the measured input function, A_m_(t) was equal to some combination of the true input function, A_t_(t), and the background activity, B(t):

A_m_(t) = a* A_t_(t) + b*B(t). equation 1

The constant "a" is less than unity because some of the counts blur out of the vessel region of interest due to the partial volume effect. Similarly "b" represents blurring of background counts into the vessel's region of interest. B(t) was only a negligibly small fraction of A(t) until late times (> 4 min) so the correction was only important at late times.

Since we were only interested in estimating the ratio of the blood input functions as a result of two different physiological challenges, air and carbogen breathing, we did not need to correct the input functions for "*a*" because the size and placement of the ROI was identical in each case:



Thus, we only needed to correct the input function for background contamination. Four venous blood samples were drawn from 30–60 min post injection. These samples were thought to be at late enough times so that venous activity concentrations would be nearly identical to arterial concentrations, so A_t_(t) = Blood(t) at late times. This was confirmed by the very slow variation of A_m_(t) versus t at these times. Therefore, for each dataset, air or carbogen, the scaling factor *b *was obtained considering the true blood activity derived from the blood draws at late time, the actual measured input function and the background activity:



To make the correction more robust, 4 time points were considered in order to estimate *b*.

For logistical reasons, blood samples could not be drawn from the same mouse. Therefore a cohort of 6 mice of the same age, size, and tumor development as the mice imaged were injected with the same volume of FDG, and each breathed either air or carbogen under the same conditions as the imaged mice. The time activity curves for all mice (n = 3, air; n = 3, carbogen) were normalized by the activities of injected ^18^F-FDG and the curves were fit temporally with a bi-exponential function. These fitted normalized blood samples were then used in the equation 3 to compute *b*. Once *b *was determined for each imaged mouse, the true arterial ratio between air and carbogen could be computed from equation 2.

### Volumes of interest

Volumes of interest around the whole tumor were defined using a semi-automatic, three-dimensional, threshold based, region-growing algorithm (MedX, Sensor Systems Inc). The threshold was based on a percentage of the average maximum intensity in the tumor. Average maximum intensity was defined as the maximum pixel in the tumor averaged with neighboring pixels in a 0.5625 mm radius sphere, to reduce statistical noise. A 30% threshold generated a VOI that visually included the entire tumor, but VOIs using other thresholds were also studied.

The very small in-plane mis-registrations of certain mice that occurred between the air and the carbogen measurements were corrected by maximizing the 3D correlation between the two image sets (FLIRT linear registration tool; Image Analysis group, Oxford University). The final series of images collected during carbogen breathing were aligned to the respective final series of images collected during air breathing. Image data outside the tumor were not used for registration. The 3D-to-3D registration was performed using six degrees of freedom (rigid body), with 3 translations and 3 rotations. After proper normalization for input function, the air data set was then subtracted from the carbogen data set, and the alignment of tumor signals was judged by eye. Although not used for alignment, the femoral vessels also appeared to be present in each data set, and the signal in those regions was zero after the subtraction, lending support to accurate registration. Registration resulted in at most a 2 mm total displacement in the region of the tumor.

## Authors' contributions

LWC, SE, JS, ALS, MVG, and JBM carried out animal imaging studies. SH, JC, and SLB conducted image analysis/modeling studies. LWC, JBM, MCK, and SLB drafted the manuscript. JBM conceived the study, and JBM, MVG, SLB, MCK, and LWC participated in the design of the study.
